# Activation and enhancement of Fredericamycin A production in deepsea-derived *Streptomyces somaliensis* SCSIO ZH66 by using ribosome engineering and response surface methodology

**DOI:** 10.1186/s12934-015-0244-2

**Published:** 2015-05-01

**Authors:** Yonghe Zhang, Huiming Huang, Shanshan Xu, Bo Wang, Jianhua Ju, Huarong Tan, Wenli Li

**Affiliations:** Key Laboratory of Marine Drugs, Ministry of Education of China, School of Medicine and Pharmacy, Ocean University of China, Qingdao, 266003 China; CAS Key Laboratory of Marine Bio-resources Sustainable Utilization, Guangdong Key Laboratory of Marine Materia Medica, RNAM Center for Marine Microbiology, South China Sea Institute of Oceanology, Chinese Academy of Sciences, 164 West Xingang Road, Guangzhou, 510301 China; State Key Laboratory of Microbial Resources, Institute of Microbiology, Chinese Academy of Sciences, Beijing, 100101 China

**Keywords:** Deepsea-derived *Streptomyces*, Ribosome engineering, Cryptic Gene cluster, Response Surface Methodology (RSM), Fredericamycin A (FDM A)

## Abstract

**Background:**

Marine microorganisms are an important source of new drug leads. However, the discovery and sustainable production of these compounds are often hampered due to the unavailable expression of cryptic biosynthetic gene clusters or limited titer. Ribosome engineering and response surface methodology (RSM) integrated strategy was developed in this study to activate cryptic gene cluster in the deepsea-derived *Streptomyces somaliensis* SCSIO ZH66, and subsequently isolation, structural analysis, and the yield enhancement of the activated compound, anticancer drug lead Fredericamycin A (FDM A), were performed.

**Results:**

In order to discover novel natural products from marine *Streptomyces* strains by genome mining strategy, the deepsea-derived *S. somaliensis* SCSIO ZH66 was subject to ribosome engineering to activate the expression of cryptic gene clusters. A resistant strain ZH66-RIF1 was thereby obtained with 300 μg/mL rifampicin, which accumulated a brown pigment with cytotoxicity on MS plate while absent in the wild type strain. After screening of fermentation conditions, the compound with pigment was purified and identified to be FDM A, indicating that the activation of a cryptic FDM A biosynthetic gene cluster was taken place in strain ZH66-RIF1, and then it was identified to be ascribed to the mutation of R444H in the β subunit of RNA polymerase. To further improve the yield efficiently, nine fermentation medium components were examined for their significance on FDM A production by Plackett–Burman design and Box-Behnken design. The optimum medium composition was achieved by RSM strategy, under which the titer of FDM A reached 679.5 ± 15.8 mg/L after 7 days of fermentation, representing a 3-fold increase compared to the original medium. In terms of short fermentation time and low-cost fermentation medium, strain ZH66-RIF1 would be an ideal alternative source for FDM A production.

**Conclusions:**

Our results would hasten the efforts for further development of FDM A as a drug candidate. Moreover, this ribosome engineering and RSM integrated methodology is effective, fast and efficient; it would be applicable to genome mining for novel natural products from other strains.

**Electronic supplementary material:**

The online version of this article (doi:10.1186/s12934-015-0244-2) contains supplementary material, which is available to authorized users.

## Introduction

After years of screening from soil dwelling microorganisms, it is more and more difficult to discover novel compounds through traditional screening strategies, which increases the drug development costs and drains the pipeline of natural product drugs. Marine microorganisms, especially *Streptomyces*, have become an important source of pharmacologically active metabolites [1,2]. Because marine environmental conditions are extremely different from the terrestrial environment [3], it is surmised that marine microorganisms have different characteristics from their terrestrial counterparts and, therefore, might produce different types of bioactive compounds. Marine *Streptomyces* are widely distributed in the oceans and, more importantly, novel compounds with biological activities have been isolated from them at a high frequency, indicating that marine *Streptomyces* are an important source for drug discovery. However, the discovery and sustainable production of bioactive compounds are often hampered due to the unavailable expression of cryptic biosynthetic gene clusters or limited incubation and testing conditions [4].

Over the past decade, actinomycete genome sequencing has revealed that many secondary metabolites biosynthetic gene clusters are “silent” under ordinary laboratory conditions [5,6], which greatly precluded discovery of novel bioactive compounds. Ribosome engineering targeting ribosomal proteins or RNA polymerase (RNAP) has been proved to be effective for waking up and upregulation of the expression of cryptic gene clusters [6-8], leading to discovery of cyclic peptide Piperidamycin A [9] and chlorinated alkaloids Inducamides A–C [10]. In our efforts to discover novel natural products from marine *Streptomyces* strains by using genome mining strategy, deepsea-derived *Streptomyces somaliensis* SCSIO ZH66 was isolated, identified and subject to ribosome engineering to activate the cryptic gene clusters in the genome. A rifampicin-resistant mutant strain accumulating dark brown pigment was obtained; the pigment was purified and identified as anticancer drug lead Fredericamycin (FDM) A.

FDM A, first isolated from terrestrial *Streptomyces griseus* ATCC49344 in 1981 [11], is an aromatic pentadecaketide featuring two sets of peri-hydroxy tricyclic aromatic moieties connected through a unique asymmetric carbaspiro center, representing a new chemotype for anticancer drug leads [12]. The cytotoxicity of FDM A against tumor cell lines resulted from inhibition of DNA topoisomerases I and II as well as the peptidyl-prolyl cis/trans isomerase Pin1, which is involved in the regulation of cell cycle and cell division [13,14]. Given its promising bioactivity and unique structural architecture, many efforts have been devoted to chemical syntheses [15-17] and biosynthesis [18-21] of FDM A. However, the limited yield of FDM A is still one of the major issues precluding its further development as an anticancer drug. Hence, the rifampicin resistant mutant strain ZH66-RIF1 may serve as an alternative efficient FDM A producer. To further improve FDM A production rapidly, response surface methodology (RSM) [22,23] was adopted. Unlike the conventional one-factor-at-a-time method, RSM is a mathematical and statistical method used to describe and predict the response of a multi-variable system with fewer experiments; therefore, it is much more efficient in terms of time and cost [24].

Here we report the ribosome engineering of *S. somaliensis* SCSIO ZH66 resulting in accumulation of dark brown pigment, the identification of the compound with pigment and its encoding gene cluster, and the rapid enhancement of FDM A production using RSM methodology.

## Results

### Ribosome engineering of a deepsea-derived *Streptomyces* strain

**Figure 1:**
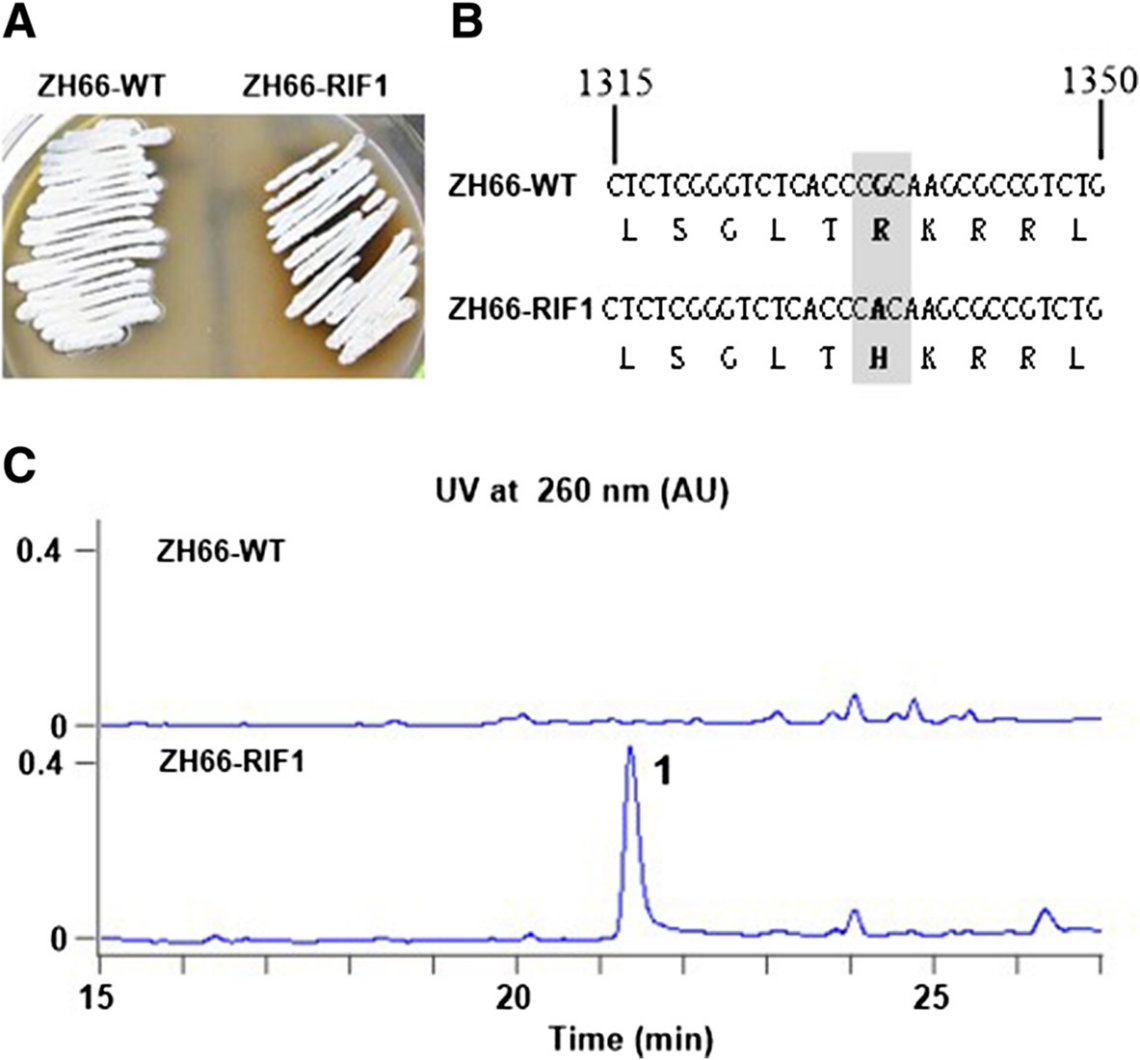
Comparisons of wild-type strain ZH66-WT and rifampicin-resistant mutant strain ZH66-RIF1. **(A)** Phenotype of strain ZH66-WT and strain ZH66-RIF1 on MS plate when incubated at 30°C for 7 days. **(B)** Comparison of the *rpoB* genes from strain ZH66-WT and strain ZH66-RIF1. **(C)** HPLC traces of strain ZH66-WT and strain ZH66-RIF1. “**1**” indicates the new compound accumulated by strain ZH66-RIF1.

*S. somaliensis* SCSIO ZH66 (CGMCC NO.9492) was isolated from the deep-sea sediment collected at a depth of 3536 m of the South China Sea. A 1520-bp 16S rRNA gene sequence of this strain was determined and compared against the EzTaxon-e server Database [25]. The result showed that it is highly similar to *Streptomyces* 16S rRNA with 99% identity to that of *Streptomyces somaliensis* (GenBank accession number AB184243). Therefore, this strain was named as *S. somaliensis* SCSIO ZH66. Phylogenetic analysis of strain ZH66 with its related *Streptomyces* type strains is shown in Additional file 1: Figure S1. Considering its unique ecological environment, the genome of strain ZH66 was sequenced, which revealed that at least 20 secondary metabolites biosynthetic gene clusters are existed in this strain (data not shown). To activate the expression of cryptic gene clusters, strain ZH66 was subject to ribosome engineering. Hence, mutant strain ZH66-RIF1 was generated as described in the Materials and methods section, which was screened at the presence of 300 μg/mL rifampicin. In contrast to the wild-type strain ZH66-WT, mutant strain ZH66-RIF1 accumulated dark brown pigment on MS plate (Figure 1A); HPLC analysis of their ethyl acetate extracts revealed production of compound **1** by strain ZH66-RIF1 but not by the wild-type strain (Figure 1C). The mutation in strain ZH66-RIF1 was then determined to be R444H in the *rpoB* gene encoding the β subunit of RNAP (Figure 1B). In addition, the morphological differentiation of strain ZH66-RIF1 was obviously impaired. As shown in Additional file 1: Figure S2, the wild-type strain ZH66-WT sporulated well on Gauze’s No.1 medium when incubated at 30°C for 72 h, while no sporulation was observed for strain ZH66-RIF1 at the same conditions. Furthermore, the extracts were tested for cytotoxicity against three human cancer cell lines. To our delight, strong inhibitions were observed for strain ZH66-RIF1 with half-effective concentration (EC_50_) of 3.4 mg/mL against A-549, 1.6 mg/mL against HCT116, and 3.9 mg/mL against P388, indicating compound **1** probably has significant anticancer bioactivity.

### Identification of FDM A production from the rifampicin resistant strain ZH66-RIF1

**Figure 2:**
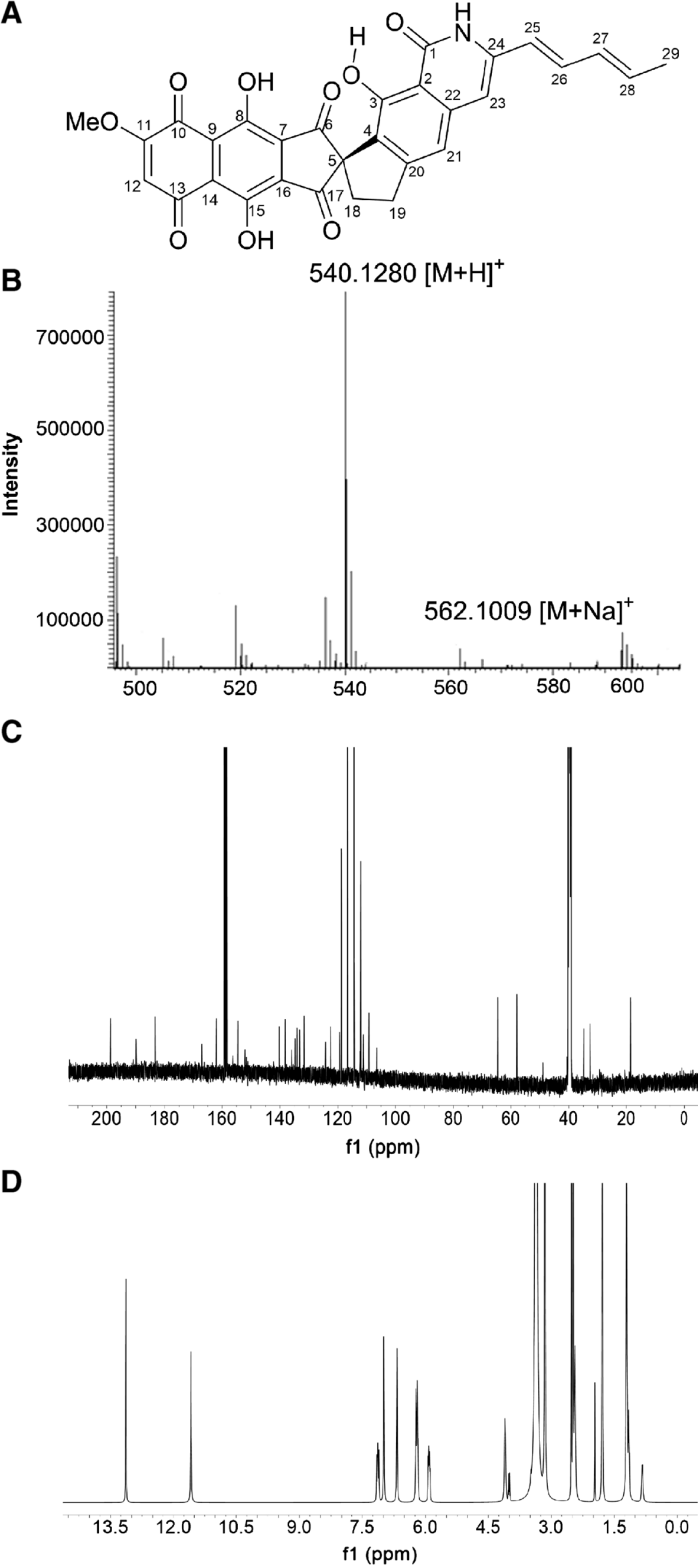
Structure determination of FDM A. **(A)** Chemical structure of FDM A. **(B)** HRMS data of FDM A. **(C)** ^1^H NMR spectrum of FDM A in DMSO-*d*_*6*_. **(D)** ^13^C NMR spectrum of FDM A in DMSO-*d*_*6*_ *.*

Given the important role of medium composition for gene expression [26], strain ZH66-RIF1 was fermented in different medium to compare the production of compound **1** (Additional file 1: Table S1); among them, medium-3 gave rise to the best yield of approximate 220.9 mg/L, which was about 30-, 5-, 10- and 13-fold higher than medium-1 (~7.2 mg/L), 2 (~42.1 mg/L), 4 (~20.9 mg/L) and 5 (~15.6 mg/L), respectively (Additional file 1: Figure S3). Therefore, fermentation (2 L) was carried out using medium-3, from which compound **1** was purified and then subject to structural analysis. The UV spectrum of compound **1** displayed λ_max_ at 260, 302, 316, 332, 357, 373 and 392 nm (Additional file 1: Figure S4), and the chemical formula of compound **1** was determined to be C_30_H_21_NO_9_ by HR-ESI-MS (m/z 540.1280 [M + H]^+^, calcd 540.1295) (Figure 2B). Both are consistent with those of FDM A [12]. The ^1^H-NMR (Figure 2C) and ^13^C-NMR (Figure 2D) data of compound **1** were further recorded, and the chemical shifts were summarized in Additional file 1: Table S2. Taken all the above data together, the identity of compound **1** was unambiguously confirmed to be the anticancer drug lead FDM A (Figure 2A). Compared with the previously reported FDM A producing strains, FDM A titers from *Streptomyces chattanoogensis* ISP 5002 [27] and *S. griseus* ATCC49344 [20] are 10 mg/L and 162 mg/L, respectively. Therefore, the strain ZH66-RIF1 may serve as an alternative producer with the FDM A titer reaching 220.9 mg/L when cultured in medium-3 (Additional file 1: Table S3).

### Identification of the FDM A biosynthetic gene cluster from strain ZH66

**Figure 3:**
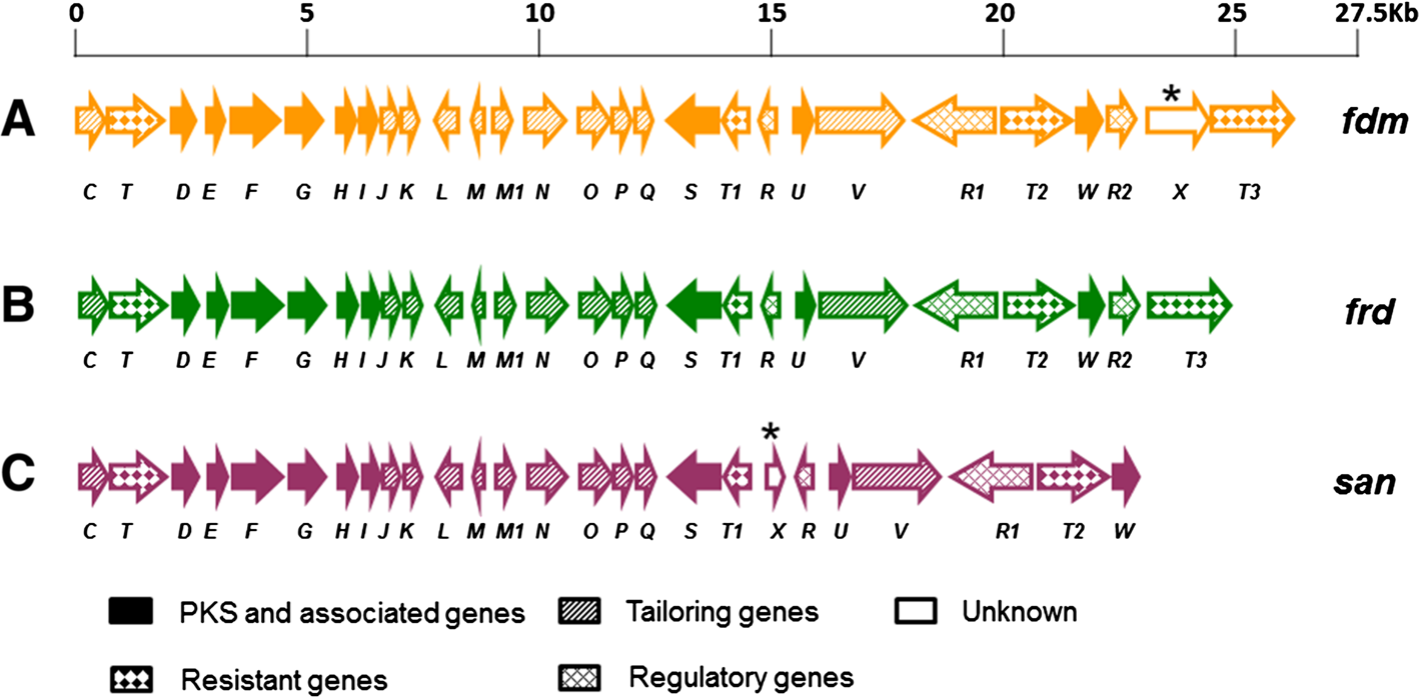
Comparative analyses of the FDM A gene clusters from different *Streptomyces* strains. **(A)** *fdm,* from *S. griseus* ATCC49344. **(B)** *frd*, from *S. somaliensis* SCSIO ZH66. **(C)** *san*, from *Streptomyces* sp. SANK61196. The unique gene was marked by a star.

**Figure 4:**
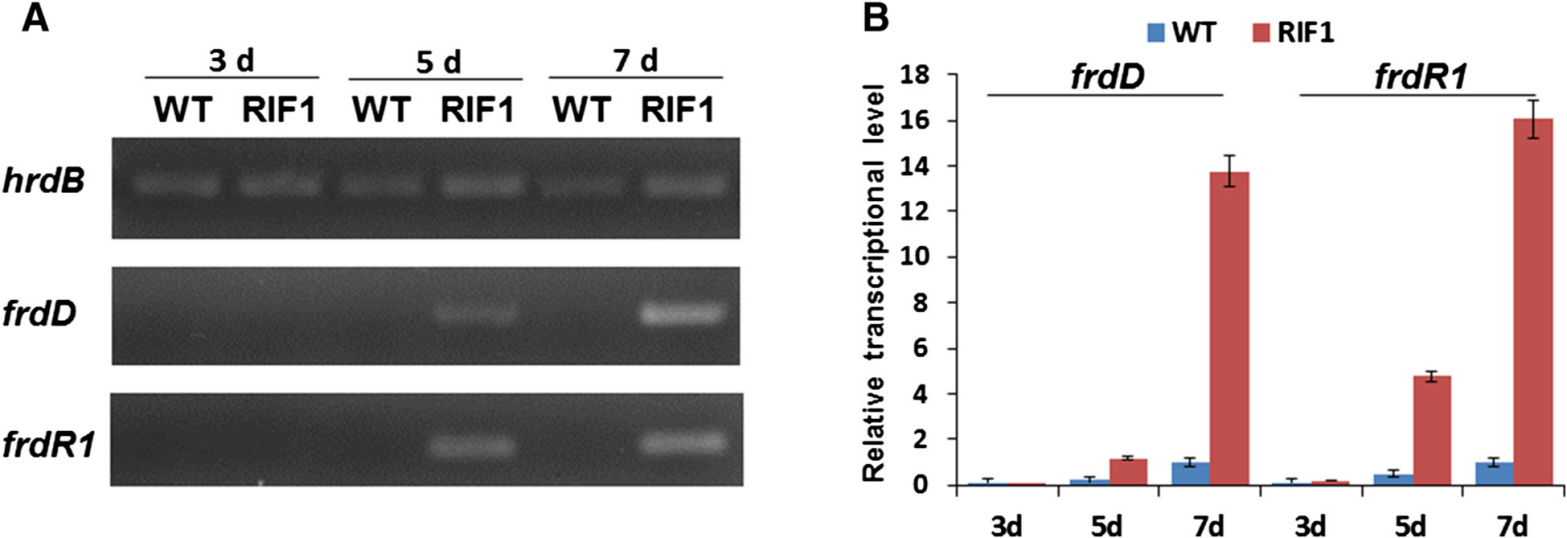
Transcriptional analysis of *frdD* and *frdR1* genes in wild-type strain ZH66-WT and rifampicin-resistant mutant strain ZH66-RIF1. Transcripts were determined by RT-PCR **(A)** and real-time RT-PCR **(B)**. The transcription levels were detected at 3, 5 and 7 days in strain ZH66-WT and strain ZH66-RIF1 grown on MS plates. Error bars indicated standard deviations (n = 3).

In consistent with the production of FDM A, genome analysis of strain ZH66 revealed the presence of a FDM gene cluster (herein named *frd*), which covers about 25-kb DNA region containing 27 open reading frames (*orfs*) with remarkably high homology to their homologs from *S. griseus* ATCC49344 (*fdm* gene cluster) [18] and *Streptomyces* sp. SANK61196 (*san* gene cluster) [28] (Additional file 1: Table S4). As shown in Figure 3, the gene composition and organization of these three clusters are highly conserved but with the following exceptions: (i) *fdmX* only exists in the *fdm* gene cluster, which putatively encodes a transposase and probably related to the horizontal transfer of the gene cluster; (ii) *sanX* with unknown function is unique for the *san* gene cluster; (iii) the counterparts of *fdmR2/frdR2* and *fdmT3/frdT3* are absent in the *san* gene cluster. The sequence was deposited in the GenBank database under the accession number KP213175. The production of FDM A in strain ZH66-RIF1 but not in strain ZH66 suggested activation of the cryptic *frd* gene cluster. The transcription levels of the *frdD* gene (encoding polyketide cyclase) and the *frdR1* gene (encoding *Streptomyces* antibiotic regulatory protein, SARP) (Additional file 1: Table S4, S5) were further analyzed by RT-PCR (Figure 4A) and real time RT-PCR (Figure 4B), confirming that both of them were substantially transcribed in strain ZH66-RIF1 in comparison with the wild-type strain ZH66.

### Screening of significant variables for FDM A production using Plackett-Burman design (PBD)

**Figure 5:**
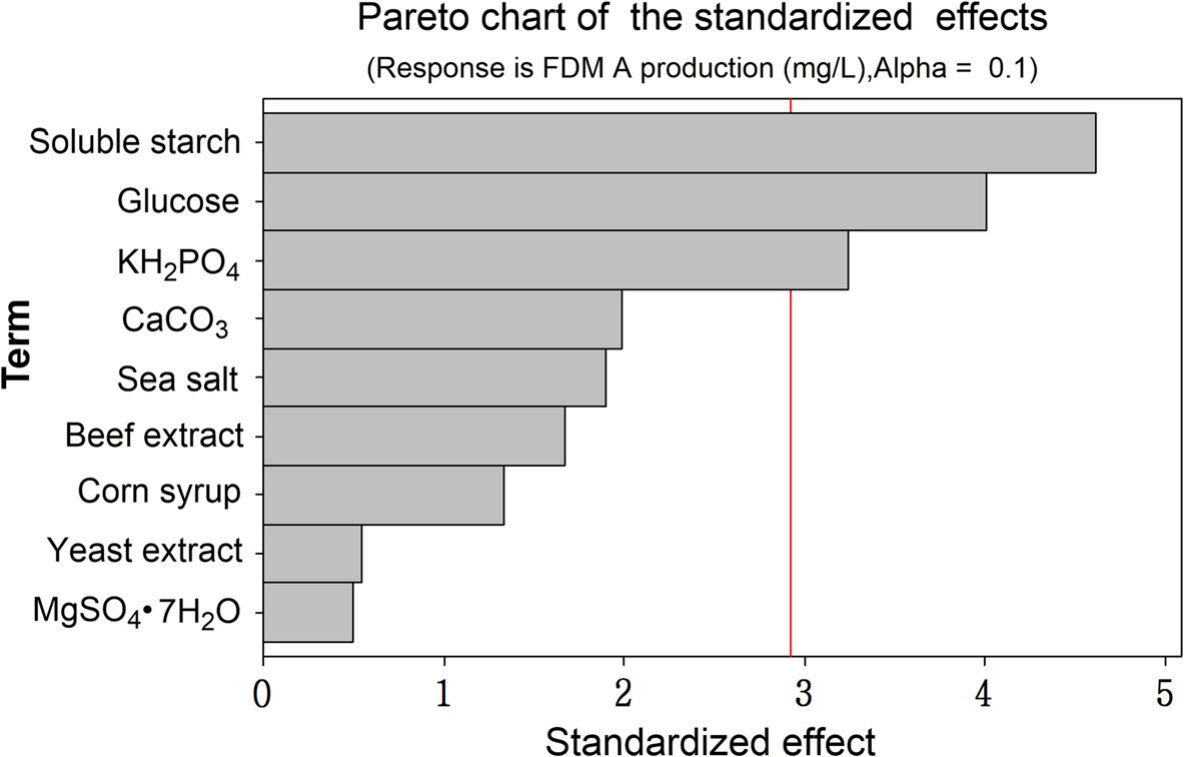
The effects of different factors on FDM A production. The bars of the diagram which go beyond the vertical red line correspond to the statistically significant effects, for a confidence level of 90%.

To further improve FDM A production efficiently, we screened the significant variables for FDM A production using PBD. As shown in Additional file 1: Table S6, nine different factors were tested, including soluble starch (*X*_*1*_), glucose (*X*_*2*_), corn syrup (*X*_*3*_), yeast extract (*X*_*4*_), beef extract (*X*_*5*_), CaCO_3_ (*X*_*6*_), MgSO_4_ · 7H_2_O (*X*_*7*_), KH_2_PO_4_ (*X*_*8*_) and sea salt (*X*_*9*_). All the experiments were performed in triplicate and the average of results (FDM A titer) were presented as the response in Additional file 1: Table S6. The effect on FDM A production was estimated for each variable (Additional file 1: Table S7). The Pareto chart (Figure 5) clearly showed that soluble starch (*X*_*1*_), glucose (*X*_*2*_), and KH_2_PO_4_ (*X*_*8*_) had confidence levels above 90% (*p* < 0.01). Therefore, these three variables were selected for further optimization.

### Optimization of FDM A production using response surface methodology

To determine the optimal levels of the above selected variables, the RSM based on Box-Behnken design (BBD) was employed. The respective coded levels for each factor were listed in Additional file 1: Table S8. The experimental design and the corresponding results were shown in Table 1. By employing multiple regression analysis on the experimental data, the following equation was obtained, describing the relationship between FDM A titer (*Y*) and the tested variables (*X*):

$$ \begin{array}{l}Y=636.19\hbox{--} 84.80{X}_1+76.81\ {X}_2\hbox{--} 66.06{X}_8\hbox{--} 65.21{X_1}^2\hbox{--} 64,543{X_2}^2\hbox{--} \\ {}\kern1.08em 35.5{X_8}^2-29.44{X}_1{X}_8+1.5{X}_1{X}_2-5.105{X}_2{X}_8\\ {}\end{array} $$

**Table 1:** **The design of experiments and response of FDM A production**

**Run**	**Soluble starch**	**Glucose**	**KH**_**2**_ **PO**_**4**_	**FDM A titer (** ***Y*** **, mg/L)**
	***X***_***1***_	***X***_***2***_	***X***_***8***_	
1	−1	−1	0	531.49 ± 5.21
2	−1	1	0	675.39 ± 20.00
3	0	1	−1	684.50 ± 8.08
4	1	−1	0	334.49 ± 19.05
5	0	1	1	548.14 ± 9.34
6	0	0	0	633.26 ± 17.38
7	0	0	0	637.19 ± 8.79
8	1	1	0	484.39 ± 3.57
9	0	−1	−1	513.95 ± 6.54
10	1	0	1	364.40 ± 4.06
11	0	−1	1	398.01 ± 7.40
12	1	0	−1	533.37 ± 3.95
13	0	0	0	628.13 ± 10.49
14	−1	0	1	578.48 ± 8.97
15	−1	0	−1	647.68 ± 8.08

Where *Y* was the predicted FDM A titer; *X*_*1*_, *X*_*2*_ and *X*_*8*_ were the concentrations of soluble starch, glucose and KH_2_PO_4_, respectively.

To validate the regression coefficient, analysis of variance (ANOVA) for FDM A production was performed. The high *F*-value (62.72) and low *p*-value (0.000 < 0.05) implied the model was highly significant (Additional file 1: Table S9). As shown in Table 2, most regression coefficients were highly significant with *p*-values less than 0.05. The regression analysis of the data showed coefficient of determination (R^2^) value of 0.9912 and adjusted R^2^ value of 0.9754, which were in close agreement. Therefore, the present R^2^ value reflected a very good fit between the observed and predicted responses, indicating that the model was reliable for FDM A production in the present study.

**Table 2:** **The least-squares fit and coefficient estimate**

**Variables**	**Coefficient estimate**	**Standard error**	***t*** **-value**	***p*** **-value**
Intercept	636.193	10.156	62.644	0.000
*X*_*1*_	−84.799	6.219	−13.635	0.000
*X*_*2*_	76.810	6.219	12.351	0.000
*X*_*8*_	−66.059	6.219	−10.622	0.000
*X*_*1*_^*2*^	−65.210	9.154	−7.124	0.001
*X*_*2*_^*2*^	−64.543	9.154	−7.051	0.001
*X*_*8*_^*2*^	−35.500	9.154	−3.878	0.012
*X*_*1*_ *X*_*2*_	1.500	8.795	0.171	0.871
*X*_*1*_ *X*_*8*_	−29.442	8.795	−3.348	0.020
*X*_*2*_ *X*_*8*_	−5.105	8.795	−0.580	0.587

R^2^ = 99.12%; R^2^adj = 97.54%.

**Figure 6:**
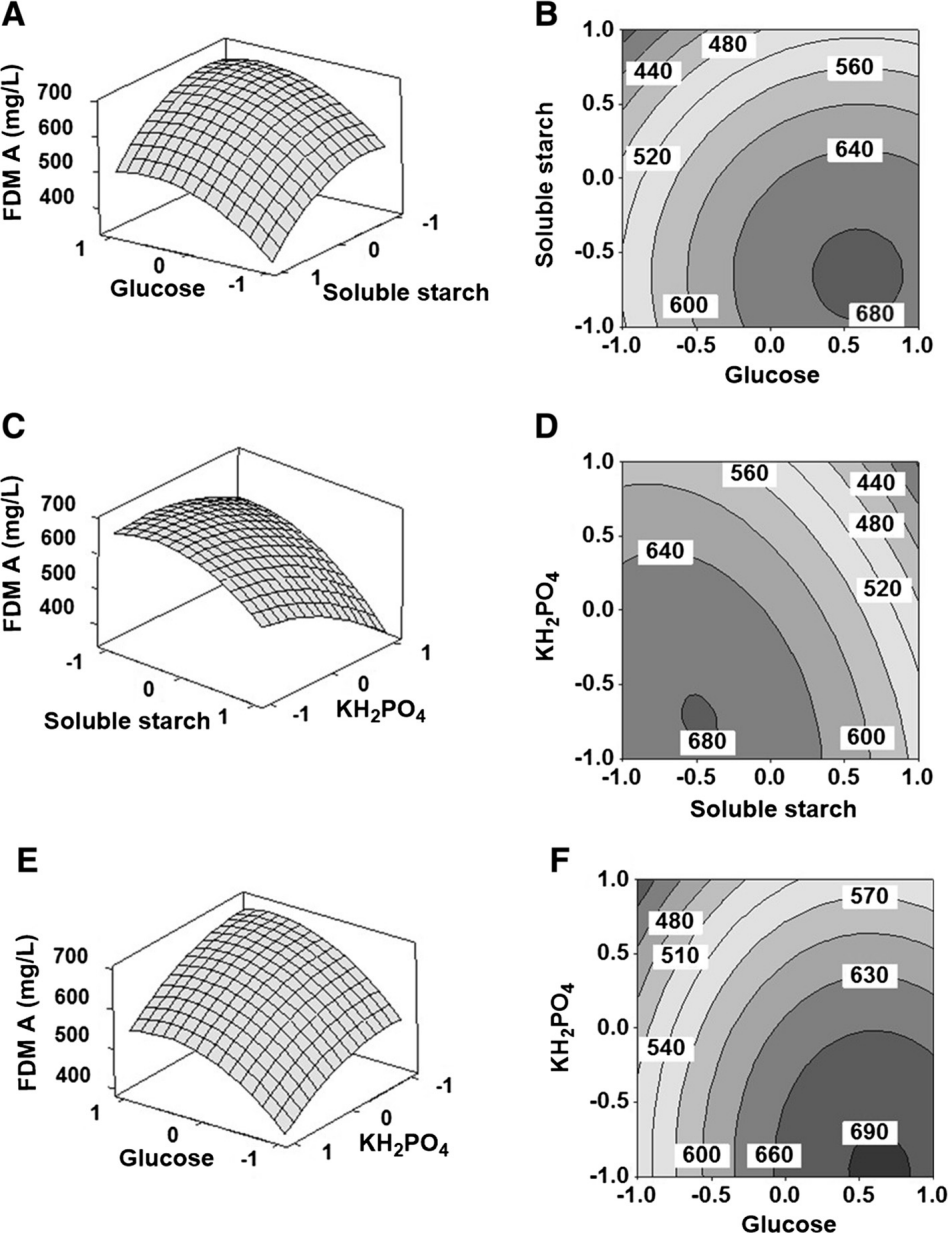
3D response surfaces and 2D contour plots for FDM A production. Each figure presented the effect of two variables on the production of FDM A, while the other one variable was held at zero level. **(A, B)** The effects of soluble starch (*X*_*1*_) and glucose (*X*_*2*_) on FDM A production. **(C, D)** The effects of soluble starch (*X*_*1*_) and KH_2_PO_4_ (*X*_*8*_) on FDM A production. **(E, F)** The effects of glucose (*X*_*2*_) and KH_2_PO_4_ (*X*_*8*_) on FDM A production.

The 3D response surfaces and 2D contour plots (Figure 6) were the graphical representations of the regression equation, which indicated the optimum ranges of the variables for the maximal response. Clearly, the FMD A production increased with increase of glucose concentration (level −1 ~ 0.6), and decrease of soluble starch (level 1 ~ −0.75) and KH_2_PO_4_ (level 1 ~ −0.7) concentrations.

### Validation of the optimized condition

Based on the above optimized medium composition, the maximum yield of FDM A was predicted to be 699.84 mg/L when the code levels of *X*_*1*_*, X*_*2*_ and *X*_*8*_ were −0.724 (soluble starch 5.55 g/L), 0.446 (glucose 27.78 g/L), and −0.706 (KH_2_PO_4_ 0.301 g/L), respectively. To confirm the predicted results, validation experiments were performed in triplicate. Under the optimal conditions, the observed experimental average concentration of FDM A was 679.5 ± 15.82 mg/L, suggesting that the experimental and the predicted values (699.84 mg/L) were in good agreement.

## Discussion

Activation of cryptic secondary metabolite biosynthetic gene clusters and improvement of production of potential drug leads are two important objects for drug discovery and development [6,7]. Ribosome engineering is an effective strategy for waking up dormant gene clusters, but the titer of the activated compounds may still be limited for further chemical and biological evaluations, which would severely hamper their development as drug candidates. In the present study, a *Streptomyces* strain was isolated from the deep sea, and was identified as *S. somaliensis* SCSIO ZH66 based on the 16S rDNA sequence (Additional file 1: Figure S1). A ribosome engineering and RSM integrated strategy was developed to activate cryptic gene clusters in strain ZH66, and subsequently enhance the titer of the activated anticancer drug lead FDM A (Figure 2), reaching a considerable yield of 679.5 ± 15.8 mg/L after 7 days of fermentation.

Ribosome engineering is performed using antibiotics that target either the ribosome itself or RNAP. Drug-resistant mutants enforced by rifampicin harbor mutations in *rpoB* gene (encoding the β subunit of RNAP) [6,7]. Recently, Fu et al. reported the discovery of chlorinated alkaloids Inducamides A–C from the rifampicin-resistant strain *Streptomyces* sp. SNC-109-M3, which was identified to contain a S442F mutation in *rpoB* gene [10]; while mutant strain ZH66-RIF1 contained a mutation of R444H, which was probably responsible for activating FDM A production (Figures 1 and 4) and reducing the growth rate of strain ZH66RIF1 as well (Additional file 1: Figure S2). Notwithstanding, additional mutations might also exist in the genome of strain ZH66-RIF1 besides the mutation in RNAP. Notably, activation of cryptic gene clusters by *rpoB* mutations was medium-dependent [9], indicating the important roles of medium composition for the expression of the activated gene clusters. Hence, strain ZH66-RIF1 was cultured under different conditions, and the yield of FDM A varied significantly in various medium (Additional file 1: Figure S3). We assume other compounds with novel structures might also produce if more culture conditions are screened. In addition, experimental tools such as HPLC-MS/MS, RT-PCR would probably be necessary to access the full profile of the activated gene clusters in strain ZH66-RIF1.

Although strain ZH66 was isolated from the deep sea, the *frd* gene cluster exhibited surprisingly high homology to those from *S. griseus* ATCC49344 and *Streptomyces* sp. SANK61196 (Figure 3, Additional file 1: Table S1). The presence of *fdmX* gene, presumably encoding a transposase, indicated that the *fdm* gene cluster in *S. griseus* ATCC49344 was probably obtained *via* horizontal gene transfer (HGT) events from other strains. Since the homology of *frd* vs *fdm* (98-100% identity/similarity on amino acids level) is much higher than that of *frd* vs *san* (62-94% identity/similarity on amino acids level), the *frd* gene cluster should have closer evolution relationship with the *fdm* gene cluster than with the *san* gene cluster. Intriguingly, in addition to FDM A, the *san* gene cluster was reported to encode a structurally distinct compound A-74528 as well [29]. Nevertheless, strain ZH66 represented the first FDM A producer isolated from marine environment. Given its promising value as anticancer drug candidate, enhancement of the FDM A titer was of interest for its further development. RSM strategy was adopted to evaluate multiple variables efficiently, leading to establishment of the optimized medium composition for FDM A production (Table 1, Figure 6). Notably, compared with the original medium, the ratio of soluble starch (slow-acting) to glucose (fast-acting) was changed from 1:2 (10 g/L to 20 g/L) to approximately 1:5 (5.55 g/L to 27.78 g/L), suggesting that low concentration of glucose probably could not meet the requirement of cell growth thus leading to low yield of FDM A.

By overexpressing the SARP-type activator gene *fdmR1* in *S. griseus* ATCC49344, the titer of FDM A reached about 997 mg/L when cultured in APM medium (medium-2, Additional file 1: Table S1) for 10 days; since a two-stage fermentation process was adopted, the total fermentation took 11 to 12 days plus 1 or 2 days for seed culture; conversely, the titer of FDM A was about only 400 mg/L on day 7 (not including seed culture) (Additional file 1: Table S3) [20]. In the present study, when cultured in APM medium, the FDM A titer was only one fifth of that in medium-3 (Additional file 1: Figure S3); a titer of 679.5 ± 15.82 mg/L was achieved in the optimized medium on day 7 in a one-stage fermentation process. In terms of the medium composition, our optimized medium is much cheaper than APM medium. Therefore, taking into account of the medium cost and fermentation time, strain ZH66-RIF1 would be an ideal FDM A producer, which could be expedient to its pharmaceutical development.

## Conclusions

Marine *Streptomyces* strains serve as important sources for novel secondary metabolites. However, many secondary metabolites biosynthetic gene clusters are “silent” in typical lab conditions. Ribosome engineering was performed to wake up the cryptic gene clusters in the deepsea-derived *S. somaliensis* SCSIO ZH66, leading to activation of a FDM biosynthetic gene cluster, which showed high homology to the ones from *S. griseus* ATCC49344 and *Streptomyces* sp. SANK61196. RSM strategy was further adopted to rapidly improve FDM A accumulation. Among the variables, soluble starch, glucose and KH_2_PO_4_ were found to be the most significant. A maximum FDM A production of 679.5 ± 15.82 mg/L was achieved with the optimized medium developed by RSM, as compared to 220.9 mg/L in the original medium. Since FDM A was a potential anticancer drug, the present study provided a new FDM A producing marine *Streptomyces* stain and a more feasible and inexpensive medium for further large scale fermentation. More importantly, ribosome engineering and RSM integrated methodology is effective, fast and efficient for activation of cryptic gene cluster and the subsequent titer optimization, which would facilitate novel compounds discovery and their sufficient production for further pharmaceutical development.

## Material and methods

### Microorganism and culture conditions

*S. somaliensis* SCSIO ZH66 (CGMCC NO.9492) was isolated from the deep sea sediment collected at a depth of 3536 meters of the South China Sea (120° 0.250′E; 20° 22.971′N). The strain was grown at 30°C on MS medium for sporulation. Five fermentation media as described in the supplemental materials (Additional file 1: Table S1) were screened to detect the FDM A production, among which medium-2 was chosen as the start medium for further optimization. For each fermentation, strain ZH66RIF-1 spores were inoculated into 50 mL of fermentation medium supplemented with 2% XAD-16 resin in a 250 mL flask, and incubated at 30°C, 240 rpm for 7 days.

### Identification of the actinomycete strain

Genomic DNA was prepared according to the literature protocol [29]. The 1520-bp 16S rRNA gene was amplified by PCR using the primer pair of 16S-FP/16S-RP (Additional file 1: Table S4). The 16S rRNA gene sequence was compared against the EzTaxon-e server Database [25] to retrieve the most similar sequences of type strains. Phylogenetic analysis of the 16S rRNA gene sequences were performed using the Molecular Evolutionary Genetics Analysis (MEGA) software [30], and the substitution model was Poisson correction. Primer synthesis and DNA sequencing were performed at Sunny Biotech Co. Ltd. (Shanghai, China).

### Ribosome engineering of strain ZH66

Firstly, the minimum inhibitory concentrations (MIC) of rifampicin against strain ZH66 were determined. Spore suspensions (10^6^ spores) were spread onto MS plates containing different concentrations (0–50 μg/mL) of rifampicin. The plates were incubated at 30°C for 5 days. The minimum concentration that fully inhibited the growth of strain ZH66 was defined as the MIC value, which turned out to be 10 μg/mL. Spore suspensions (10^9^ spores) were then spread onto MS plates containing rifampicin at concentrations of 30 to 300 μg/mL. Mutant colonies producing the darkest pigment were selected, generating mutant strain ZH66-RIF1 which was obtained on the MS plate containing 300 μg/mL rifampicin. To map the mutation in the genome of strain ZH66RIF-1, the *rpoB* gene was amplified using Q5 high-fidelity DNA polymerase (New England Biolabs, Beverly, MA, USA) with the primer pair of rpoB-FP/rpoB-RP (Additional file 1: Table S4). The amplified DNA fragment was digested with *Bam*HI and *Hin*dIII, and then was ligated into the same sites of pUC18 to yield pUC18::*rpoB*-RIF1. After confirmation by enzymatic digestion, three clones were subject to sequencing to analyze the mutation in *rpoB* gene.

### Genomic scanning and sequence analysis

Genomic DNA was shotgun sequenced using commercial 454 technology by Shanghai Majorbio Bio-pharm Technology Co., Ltd. (Shanghai, China). *orf* assignments and their proposed function were accomplished by using the FramePlot 4.0beta (http://nocardia.nih.go.jp/fp4) [31] and Blast programs (http://blast.ncbi.nlm.nih.gov/Blast.cgi) [32], respectively.

### Biological assays

Cytotoxicity assays were performed at the Molecular Screening Facility at School of Pharmacy, Ocean University of China (Qingdao, China), by using the SRB method (against A-549 and HCT116), or the MTT method (against P388).

### Production and analyses of FDM A

The fermentation cultures were harvested by centrifugation, and the supernatant was extracted twice with an equal volume of ethyl acetate. The combined EtOAc extracts were concentrated *in vacuo* to afford residue A. The precipitated mycelia and XAD-16 resin were extracted twice with acetone. The extracts were combined, and acetone was evaporated *in vacuo* to yield residue B. The combined residues were dissolved in 95:5 mixture of CHCl_2_-MeOH, filtered through a 0.2 μm filter, and subject to HPLC. The HPLC system consisted of Agilent 1260 Infinity Quaternary pumps and a 1260 Infinity diode-array detector. Analytical HPLC was performed on an Eclipse C18 column (5 μm, 4.6 × 150 mm) developed with a linear gradient from 65% to 100% B/A (phase A: 0.1% formic acid in H_2_O; phase B: 100% acetonitrile supplemented with 0.1% formic acid) in 20 min followed by an additional 10 min at 100% B at flow rate of 1 mL/min and UV detection at 260 nm. For FDM A purification, semi-preparative HPLC was carried out using an YMC-Pack ODS-A C18 column (5 μm, 120 nm, 250 × 10 mm). Samples were eluted with a linear gradient from 50% to 95% B/A in 40 min, followed by 100% B for 10 min at a flow rate of 2.0 mL/min and UV detection at 260 nm. The identity of FDM A was confirmed by HRMS and NMR analysis. HRMS was carried out on Thermo LTQ-XL mass spectrometer. NMR data was recorded with a Bruker Avance 600 spectrometer.

### Transcriptional analysis by RT-PCR and real-time RT-PCR

Total RNAs were prepared using Ultrapure RNA Kit (CWBio. Inc., Beijing, China). The genomic DNA was removed with gDNA Eraser (Takara Bio, Inc., Dalian, China). Synthesis of the first strand cDNA was performed with PrimeScript RT reagent Kit (Takara Bio, Inc., Dalian, China). The reaction parameters of reverse transcription PCR (RT-PCR) were as follows: 95°C for 5 min, followed by 30 cycles consisting of 95°C for 30 s, 60°C for 30 s, 72°C for 45 s and a final extension of 72°C for 10 min. Quantitative real-time reverse transcription PCR (real-time RT-PCR) was performed on an ABI7500 real-time PCR system (Applied Biosystems) in triplicate for each sample using the SYBR green method. Each reaction mixture contained 5 μL of SYBR Premix Ex Taq (Takara Bio, Inc., Dalian, China), 0.2 μL of ROX Reference Dye II (50×), 0.5 μL of 2.5-fold diluted cDNA, 0.2 μL of each primer at a concentration of 0.2 pmol μL^−1^ (Additional file 1: Table S4) and 3.4 μL ddH_2_O. The PCR procedures were as follows: 95°C for 10 min, 40 cycles 95°C for 15 s and 60°C for 1 min. The *hrdB* gene (Additional file 1: Table S4) was used as the reference for RT-PCR and real-time RT-PCR.

### Identifying the significant variables using Plackett-Burman design

Preliminary investigation of factors affecting the FDM A production was done by using PBD [33]. The variables chosen for the present study were investigated at two widely spaced intervals designated as −1 (low level) and +1 (high level). The experimental design for screening the medium components was shown in Additional file 1: Table S6. The effects of individual parameters on FDM A production were calculated by the following equation:

$$ \mathrm{E} = \left({\displaystyle \sum {\mathrm{M}}^{+}}\hbox{-} {\displaystyle \sum {\mathrm{M}}^{\hbox{-} }}\right)\ /\ \mathrm{N} $$

Where, E is the effect of parameter under study and M^+^ and M^−^ are responses (FDM A titer) of trials at which the parameter was at its higher and lower levels, respectively, and N is the total number of trials.

### Optimization by Box-Behnken design

Levels of the significant parameters and the interaction effects between various media constituents, which significantly influence FDM A production, were analyzed and optimized by BBD [34,35]. In this study, the experimental plan consisted of 15 trials and the independent variables were studied at three different levels (−1, 0, 1). All the experiments were done in triplicate and the average of FDM A production obtained was taken as the response (*Y*). The second order polynomial coefficients were calculated and analyzed using Minitab16. The general form of the second degree polynomial equation is:

$$ Y={\beta}_0+{\displaystyle \sum {\beta}_i}{X}_i+{\displaystyle \sum {\beta}_{ii}}{X^2}_i+{\displaystyle \sum {\beta}_{ij}}{X}_i{X}_j $$

Where, *Y* is the predicted response, *X*_*i*_*X*_*j*_ are input variables which influence the response variable *Y*; *β*_*0*_ is the offset term; *β*_*i*_ is the _*i*_th linear coefficient; *β*_*ii*_ is the _*i*_th quadratic coefficient and *β*_*ij*_ is the _*ij*_th interaction coefficient. Statistical analysis of the model was performed in the form of ANOVA. This analysis included the Fisher’s *F*-test (overall model significance), its associated probability *P*(*F*), correlation coefficient R, and determination coefficient R^2^ which measures the goodness of fit of regression model. For each variable, the quadratic models were represented as counter plots and response surface curves which were generated using Minitab16.

### Software for experimental design

The software Minitab16 was used for generation and evaluation of the statistical experimental design. The optimal medium composition for FDM A production was obtained by solving the regression equation and by analyzing the response surface contour plots using the same software.

### Nucleotide sequence accession number

The nucleotide sequences of the 16S rRNA gene, the *rpoB* gene and the *frd* gene cluster reported in this paper have been deposited in the GenBank database under accession numbers of KB146142, KP722021 and KP213175, respectively.

## Additional file

Additional file 1: Table S1. Fermentation media used for detecting FDM A production by strain ZH66-RIF1. **Table S2.** ^1^H and ^13^C NMR data of FDM A in DMS0-*d*_*6*_ *.* **Table S3.** FDM A titers from different producing strains. **Table S4.** The *frd* biosynthetic gene cluster from *S. somaliensis* SCSIO ZH66. **Table S5.** The primer pairs used in this study. **Table S6.** Screening of significant variables for FDM A production using Plackett-Burman Design (PBD). **Table S7.** The effects of each factor on FDM A production. **Table S8.** The dose of important factors in response surface analysis. **Table S9.** Analysis of variance (ANOVA) for the second-order polynomial model. **Figure S1.** Phylogenetic tree of *S. somaliensis* SCSIO ZH66 based on 16S rRNA sequences. **Figure S2.** Phenotypes of strain ZH66-WT and strain ZH66-RIF1. **Figure S3.** HPLC traces of fermentation products of strain ZH66-RIF1 in different medium. **Figure S4.** UV spectrum of FDM A.

## References

[1] Jensen PR, Gontang E, Mafnas C, Mincer TJ, Fenical W (2005). Culturable marine actinomycete diversity from tropical Pacific Ocean sediments. Environ Microbiol.

[2] Lam KS (2007). New aspects of natural products in drug discovery. Trends Microbiol.

[3] Fenical W, Jensen PR (2006). Developing a new resource for drug discovery: marine actinomycete bacteria. Nat Chem Biol.

[4] Hertweck C (2009). Hidden biosynthetic treasures brought to light. Nat Chem Biol.

[5] Hopwood DA. (2008) Small things considered: the tip of the iceberg. http://schaechter.asmblog.org/schaechter/2008/06/the-tip-of-the.html.

[6] Ochi K, Hosaka T (2013). New strategies for drug discovery: activation of silent or weakly expressed microbial gene clusters. Appl Microbiol Biot.

[7] Pan Y, Lu C, Dong H, Yu L, Liu G, Tan H (2013). Disruption of *rimP-SC*, encoding a ribosome assembly cofactor, markedly enhances the production of several antibiotics in *Streptomyces coelicolor*. Microb Cell Fact.

[8] Ochi K, Tanaka Y, Tojo S (2014). Activating the expression of bacterial cryptic genes by *rpoB* mutations in RNA polymerase or by rare earth elements. J Ind Microbiol Biot.

[9] Hosaka T, Ohnishi-Kameyama M, Muramatsu H, Murakami K, Tsurumi Y, Kodani S (2009). Antibacterial discovery in actinomycetes strains with mutations in RNA polymerase or ribosomal protein S12. Nat Biotechnol.

[10] Fu P, Jamison M, La S, MacMillan JB (2014). Inducamides A–C, Chlorinated alkaloids from an RNA polymerase mutant strain of *Streptomyces* sp. Org Lett.

[11] Pandey RC, Toussaint MW, Stroshane RM, Kalita C, Aszalos AA, Garretson AL (1981). Fredericamycin A, a new antitumor antibiotic. I. Production, isolation and physicochemical properties. J Antibiot.

[12] Misra R, Pandey RC, Hilton BD, Roller PP, Silverton JV (1987). Structure of fredericamycin A, an antitumor antibiotic of a novel skeletal type; spectroscopic and mass spectral characterization. J Antibiot.

[13] Liou Y-C, Ryo A, Huang H-K, Lu P-J, Bronson R, Fujimori F (2002). Loss of Pin1 function in the mouse causes phenotypes resembling cyclin D1-null phenotypes. Proc Natl Acad Sci U S A.

[14] Latham MD, King CK, Gorycki P, Macdonald TL, Ross WE (1989). Inhibition of topoisomerases by fredericamycin A. Cancer Chemoth Pharm.

[15] Clive D, Angoh AG, Bennett SM (1987). Radical spirocyclization: synthesis of an appropriately oxygenated spiro compound related to the antitumor antibiotic fredericamycin A. J Org Chem.

[16] Clive DL, Tao Y, Khodabocus A, Wu Y, Angoh AG, Bennett SM (1994). Total synthesis of crystalline (±)-fredericamycin A. Use of radical spirocyclization. J Am Chem Soc.

[17] Boger DL, Hueter O, Mbiya K, Zhang M (1995). Total synthesis of natural and ent-fredericamycin A. J Am Chem Soc.

[18] Wendt-Pienkowski E, Huang Y, Zhang J, Li B, Jiang H, Kwon H (2005). Cloning, sequencing, analysis, and heterologous expression of the fredericamycin biosynthetic gene cluster from *Streptomyces griseus*. J Am Chem Soc.

[19] Chen Y, Luo Y, Ju J, Wendt-Pienkowski E, Rajski SR, Shen B (2008). Identification of fredericamycin E from *Streptomyces griseus*: Insights into fredericamycin A biosynthesis highlighting carbaspirocycle formation. J Nat Prod.

[20] Chen Y, Wendt-Pienkowski E, Shen B (2008). Identification and utility of FdmR1 as a *Streptomyces* antibiotic regulatory protein activator for fredericamycin production in *Streptomyces griseus* ATCC 49344 and heterologous hosts. J Bacteriol.

[21] Chen Y, Wendt-Pienkoski E, Rajski SR, Shen B (2009). In vivo investigation of the roles of FdmM and FdmM1 in fredericamycin biosynthesis unveiling a new family of oxygenases. J Biol Chem.

[22] Hunter WG, Koehler TL, Juran JM, Gryna FM (1979). Response surface methodology. Quality control handbook.

[23] Wells ME, Powers JJ, Moskowitz HR (1976). Response surface methodology and subjective data. Correlating sensory objective measurements-new methods for answering old problems.

[24] Bezerra MA, Santelli RE, Oliveira EP, Villar LS, Escaleira LA (2008). Response surface methodology (RSM) as a tool for optimization in analytical chemistry. Talanta.

[25] Kim OS, Cho YJ, Lee K, Yoon SH, Kim M, Na H (2012). Introducing EzTaxon-e: a prokaryotic 16S rRNA gene sequence database with phylotypes that represent uncultured species. Int J Syst Evol Micr.

[26] Tanaka Y, Kasahara K, Hirose Y, Murakami K, Kugimiya R, Ochi K (2013). Activation and products of the cryptic secondary metabolite biosynthetic gene clusters by rifampin resistance (*rpoB*) mutations in actinomycetes. J Bacteriol.

[27] Hosoya Y, Okamoto S, Muramatsu H, Ochi K (1998). Acquisition of certain streptomycin-resistant (*str*) mutations enhances antibiotic production in bacteria. Antimicrob Agents Chemother.

[28] Zaleta-Rivera K, Charkoudian LK, Ridley CP, Khosla C (2010). Cloning, sequencing, heterologous expression, and mechanistic analysis of A-74528 biosynthesis. J Am Chem Soc.

[29] Kieser T, Bibb MJ, Buttner MJ, Chater KF, Hopwood DA (2000). Practical *Streptomyces* Genetics.

[30] Tamura K, Stecher G, Peterson D, Filipski A, Kumar S (2013). MEGA6: molecular evolutionary genetics analysis version 6.0. Mol Biol Evol.

[31] Ishikawa J, Hotta K (1999). FramePlot: a new implementation of the frame analysis for predicting protein‐coding regions in bacterial DNA with a high G + C content. FEMS Microbiol Lett.

[32] Altschul SF, Gish W, Miller W, Myers EW, Lipman DJ (1990). Basic local alignment search tool. J Mol Biol.

[33] Tyssedal J, Ruggeri F, Kenett RS, Faltin FW (2008). Plackett–Burman designs. Encyclopedia of statistics in quality and reliability.

[34] Kalil S, Maugeri F, Rodrigues M (2000). Response surface analysis and simulation as a tool for bioprocess design and optimization. Process Biochem.

[35] Gilmour SG (2006). Response surface designs for experiments in bioprocessing. Biometrics.

