# Supplementary material for: Activation and enhancement of Fredericamycin A production in deepsea-derived Streptomyces somaliensis SCSIO ZH66 by using ribosome engineering and response surface methodology
Source: Microb Cell Fact. 2015 May 1;14:64. doi: 10.1186/s12934-015-0244-2 (PMC4425903; doi:10.1186/s12934-015-0244-2)
Supplement: Additional file 1: Table S1. — Fermentation media used for detecting FDM A production by strain ZH66-RIF1. Table S2. 1H and 13C NMR data of FDM A in DMS0-d 6 . Table S3. FDM A titers from different producing strains. Table S4. The frd biosynthetic gene cluster from S. somaliensis SCSIO ZH66. Table S5. The primer pairs used in this study. Table S6. Screening of significant variables for FDM A production using Plackett-Burman Design (PBD). Table S7. The effects of each factor on FDM A production. Table S8. The dose of important factors in response surface analysis. Table S9. Analysis of variance (ANOVA) for the second-order polynomial model. Figure S1. Phylogenetic tree of S. somaliensis SCSIO ZH66 based on 16S rRNA sequences. Figure S2. Phenotypes of strain ZH66-WT and strain ZH66-RIF1. Figure S3. HPLC traces of fermentation products of strain ZH66-RIF1 in different medium. Figure S4. UV spectrum of FDM A. [file 12934_2015_244_MOESM1_ESM.pdf]

Supporting Information

Activation and enhancement of Fredericamycin A production in deepsea-derived *Streptomyces somaliensis* SCSIO ZH66 by using ribosome engineering and response surface methodology

Yonghe Zhang[1†], Huiming Huang[1†], Shanshan Xu[1], Bo Wang[2], Jianhua Ju[2], Huarong Tan[3] and Wenli Li[1]*

[1] Key Laboratory of Marine Drugs, Ministry of Education of China, School of Medicine and Pharmacy, Ocean University of China, Qingdao 266003, China

[2] CAS Key Laboratory of Marine Bio-resources Sustainable Utilization, Guangdong Key Laboratory of Marine Materia Medica, RNAM Center for Marine Microbiology, South China Sea Institute of Oceanology, Chinese Academy of Sciences, 164 West Xingang Road, Guangzhou 510301, China

[3] State Key Laboratory of Microbial Resources, Institute of Microbiology, Chinese Academy of Sciences, Beijing 100101, China

[†] These authors contributed equally to this work.

* Author to whom correspondence should be addressed

Email addresses:

YZ: zhangyonghe1990@163.com

HH: hmhuang1988@163.com

XS: xushanshan328@163.com

BW: wangbo@scsio.ac.cn

JJ: jju@scsio.ac.cn

TH: tanhr@im.ac.cn

WL: liwenli@ouc.edu.cn

Table of contents

No.		Page
1.	**Table S1** Fermentation media used for detecting FDM A production by strain ZH66-RIF1.	S3
2.	**Table S2** ^{1}H and ^{13}C NMR data of FDM A in DMS0-d_6.	S4
3.	**Table S3** FDM A titers from different producing strains.	S5
4.	**Table S4** The *frd* biosynthetic gene cluster from *S. somaliensis* SCSIO ZH66.	S6
5.	**Table S5** The primer pairs used in this study.	S7
6.	**Table S6** Screening of significant variables for FDM A production using Plackett-Burman Design (PBD).	S7
7.	**Table S7** The effects of each factor on FDM A production.	S8
8.	**Table S8** The dose of important factors in response surface analysis.	S8
9.	**Table S9** Analysis of variance (ANOVA) for the second-order polynomial model.	S8
10.	**Figure S1** Phylogenetic tree of *S. somaliensis* SCSIO ZH66 based on 16S rRNA sequences.	S9
11.	**Figure S2** Phenotypes of strain ZH66-WT and strain ZH66-RIF1.	S9
12.	**Figure S3** HPLC traces of fermentation products of strain ZH66-RIF1 in different medium.	S10
13	**Figure S4** UV spectrum of FDM A.	S10
14	Reference	S11

Table S1 Fermentation media used for detecting FDM A production by strain ZH66-RIF1.

Name	Recipe
1.medium-1	peptone 2 g/L, yeast extract 5 g/L, soluble starch 2 g/L, soy flour 15 g/L, NaCl 4 g/L, $CaCO_3$ 4 g/L, sea salt 33 g/L, pH=7.0
2.medium-2 (APM)	yeast extract 8 g/L, malt extract 20 g/L, NaCl 2g/L, MOPS sodium salt 10 g/L in 500 mL of tap water and 380 mL of Milli-Q water. After autoclaving, 1 mL of 10% $MgSO_4$, 1 mL of 1% $FeSO_4$, 0.1 mL of 10% $ZnSO_4$ and 120 mL of 50% glucose were added [1]
3.medium-3	soluble starch 10 g/L, glucose 20 g/L, corn syrup 4 g/L, yeast extract 10 g/L, beef extract 3 g/L, $MgSO_4{\bullet}7H_2O$ 0.5 g/L, KH_2PO_4 0.5 g/L, $CaCO_3$ 2 g/L, sea salt 30 g/L， pH=7.0
4.medium-4	soluble starch 20 g/L, soy flour 5 g/L, yeast extract 5 g/L, peptone 2 g/L, NaCl 4 g/L, $CaCO_3$ 2 g/L, sea salt 33 g/L， pH=7.2~7.4
5.medium-5	malt extraction powder 40 g/L, yeast extract powder 4 g/L, glucose 20 g/L, $(NH_4)_2HPO_4$ 1 g/L， pH=7.0

Table S2 ^{1}H and ^{13}C NMR data of FDM A in DMS0-d_6.

Position	δ_C	δ_H (multiplicity, *J*, Hz)
1	167.17	
2	111.11	
3	156.35	
4	124.31	
5	64.65	
6	198.79	
7	134.80	
8	152.08	
9	118.87	
10	183.42	
11	162.02	
OCH_3-11	57.92	3.99 (3H)
12	112.26	
13	189.93	
14	109.18	
15	151.68	
16	134.20	
17	198.82	
18	34.86	2.47(t, *J* = 7.5, 2H)
19	32.69	3.15(t, *J* = 7.5, 2H)
20	154.61	
21	112.26	6.99 (s, 1H)
22	140.34	
23	106.55	6.68 (s, 1H)
24	138.30	
-NH		11.58 (s, 1H)
25	122.62	6.20 (ddq, *J* = 15.4, 8.9 Hz, 1H)
26	133.29	7.13 (dd, *J* = 15.8, 10.4 Hz, 1H)
27	131.69	6.23(d, *J* = 15.4, 1H)
28	138.30	5.91 (dd, *J* = 14.8, 7.2 Hz, 1H)
29	18.60	1.79 (d, *J* = 6.8 Hz, 3H)
OH-3		13.13 (s, 1H)

Table S3. FDM A titers from different producing strains

Strain	Culture conditions			Titer (mg/L)
	Seed medium	Production medium	Time (days)	
S. chattanoogensis ISP 5002 [2]	galactose -glycerol-corn steep	L-phenylalanine 5 g/L, $(NH_4)_2SO_4$ 1.5 g/L, K_2HPO_4 0.5 g/L, KH_2PO_4 0.5 g/L, $CaCO_3$ 2 g/L, pH=7.0	6	10
S. griseus ATCC49344 [3]	R2YE	APM (medium-2)	9-10	162
S. griseus SB4012 [3]			11-12	997
			8-9	400
S. *somaliensis* SCSIO ZH66-RIF1	-	medium-3	7	220.9
	-	optimized medium-3	7	679.5

Table S4 Proposed functions of proteins encoded by the *frd* biosynthetic gene cluster from *S. somaliensis* SCSIO ZH66 and its comparison with other FDM A gene clusters.

SCSIO ZH66			Comparison of ant gene cluster from other strains					
			Streptomyces griseus			*Streptomyces* sp. SANK 61196		
Protein	Size (aa)	Proposed function	protein	Accession number	Identity (%) /Similarity	protein	Accession number	Identity (%) /Similarity
FrdC	246	putative 3-ketoacyl-ACP reductase	FmdC	AAQ08912	99/99	SanC	ADG86311	83/90
FrdT	493	putative peptide transporter	FmdT	AAQ08913	99/99	SanT	ADG86312	82/89
FrdD	106	putative polyketide cyclase	FmdD	AAQ08914	100/100	SanD	ADG86313	89/94
FrdE	141	putative polyketide cyclase	FmdE	AAQ08915	100/100	SanE	ADG86314	83/88
FrdF	416	3-ketoacyl-ACP synthase	FmdF	AAQ08916	99/99	SanF	ADG86315	92/96
FrdG	403	chain length factor	FmdG	AAQ08917	99/99	SanG	ADG86316	84/90
FrdH	84	ACP	FmdH	AAQ08918	100/100	SanH	ADG86317	80/89
FrdI	155	putative polyketide cyclase	FmdI	AAQ08919	99/100	SanI	ADG86318	83/89
FrdJ	101	putative monooxygenase	FmdJ	AAQ08920	99/100	SanJ	ADG86319	73/81
FrdK	142	putative oxygenase	FmdK	AAQ08921	99/100	SanK	ADG86320	80/87
FrdL	246	putative monooxygenase	FmdL	AAQ08922	100/100	SanL	ADG86321	78/87
FrdM	148	putative monooxygenase	FmdM	AAQ08923	99/100	SanM	ADG86322	83/89
FrdM1	154	putative monooxygenase	FmdM1	AAQ08924	99/99	SanM1	ADG86323	81/87
FrdN	320	putative o-methyltransferase	FmdN	AAQ08925	98/99	SanN	ADG86324	78/84
FrdO	249	putative 3-ketoacyl-ACP reductase	FmdO	AAQ08926	99/100	SanO	ADG86325	88/94
FrdP	98	putative monooxygenase	FmdP	AAQ08927	99/100	SanP	ADG86326	81/92
FrdQ	105	putative monooxygenase	FmdQ	AAQ08928	99/100	SanQ	ADG86327	74/83
FrdS	333	putative 3-keto-acyl-ACP synthase	FmdS	AAQ08929	99/99	SanS	ADG86328	86/92
FrdT1	302	putative membrane transporter	FmdT1	AAQ08930	100/100	SanT1	ADG86329	87/93
-	-	-	-	-	-	SanX	ADG86330	-
FrdR	151	transcriptional regulator	FmdR	AAQ08931	100/100	SanR	ADG86331	80/90
FrdU	141	unknown	FmdU	AAQ08932	99/99	SanU	ADG86332	62/76
FrdV	622	asparagine synthetase	FmdV	AAQ08933	99/99	SanV	ADG86333	78/83
FrdR1	580	regulatory protein	FmdR1	AAQ08934	99/100	SanR1	ADG86334	73/81
FrdT2	531	putative membrane transporter	FmdT2	AAQ08935	99/99	SanT2	ADG86335	78/83
FrdW	159	holo-ACP synthase	FmdW	AAQ08936	99/99	SanW	ADG86336	69/75
FrdR2	170	transcriptional regulator	FmdR2	AAQ08937	99/99	-	-	-
-	-	-	FmdX	AAQ08938	-	-	-	-
FrdT3	525	putative membrane transporter	FmdT3	AAQ08939	99/99	-	-	-

-: means not available.

Table S5. The primer pairs used in this study.

Name	Sequence (5'-3') [a]
16S-FP	AGAGTTTGATCCTGGCTCAG
16S-RP	AAGGAGGTGATCCAGCCGCA
rpoB-FP[a]	CGGGATCCTTGGCCGCCTCGCGCACT
rpoB-RP[a]	CCCAAGCTTTCAGACCTCTTCGACGCT
hrdB-FP	CGACTACACCAAGGGCTACAA
hrdB-RP	GGAGCATCTGACGCTGGAC
frdD-FP	GGAGCCGGGTGTTGATGT
frdD-RP	GACGACGCCGACAAGGTG
frdR1-FP	GCACCGAAGGTCCGACAAGTT
frdR1-RP	CGCAGAGTCCGGGCAGTTT

[a]Underlined letters represent restriction sites.

Table S6 Screening of significant variables for FDM A production using Plackett-Burman Design (PBD)

Treatments	Variable Level									FDM A titer (mg/L)
	X_1	X_2	X_3	X_4	X_5	X_6	X_7	X_8	X_9	
1	-1	1	1	-1	-1	-1	1	1	1	436.57±11.85
2	-1	-1	-1	-1	-1	-1	-1	-1	-1	522.02±6.24
3	1	1	1	-1	1	1	-1	1	-1	308.29±24.23
4	-1	-1	-1	1	1	1	-1	1	1	373.82±13.66
5	-1	1	-1	-1	1	1	1	-1	1	481.57±26.23
6	-1	-1	1	1	1	-1	1	1	-1	385.65±15.51
7	1	-1	1	-1	1	-1	-1	-1	1	252.64±4.92
8	-1	1	1	1	-1	1	-1	-1	-1	563.54±11.04
9	1	1	-1	1	1	-1	1	-1	-1	507.76±14.34
10	1	1	-1	1	-1	-1	-1	1	1	346.15±5.80
11	1	-1	1	1	-1	1	1	-1	1	242.87±16.44
12	1	-1	-1	-1	-1	1	1	1	-1	174.59±13.76

X_1, soluble starch at a low level (-1) of 8 g/L and a high level (+1) of 12 g/L; X_2, glucose at a low level (-1) of 16 g/L and a high level (+1) of 24 g/L; X_3, at a low level (-1) of 3.2 g/L and a high level (+1) of 4.8 g /L; X_4, at a low level (-1) of 8 g/L and a high level (+1) of 12 g/L; X_5, at a low level (-1) of 2.4 g/L and a high level (+1) of 3.6 g/L; X_6, at a low level (-1) of 2.4 g/L and a high level (+1) of 3.6 g/L; X_7, at a low level (-1) of 0.4 g/L and a high level (+1) of 0.6 g/L; X_8, at a low level (-1) of 0.4 g/L and a high level (+1) of 0.6 g/L; X_9, at a low level (-1) of 24 g/L and a high level (+1) of 36 g/L.

Table S7 The effects of each factor on FDM A production.

Factor signs	Factor	Effect	Coefficient	*t* Value	*p* Value
X_1	Starch soluble	-148.64	-74.32	-4.62	0.044
X_2	Glucose	128.88	64.44	4.00	0.057
X_3	Corn syrup	-42.89	-21.45	-1.33	0.314
X_4	Yeast extract	17.50	8.75	0.54	0.641
X_5	Beef extract	53.85	26.93	1.67	0.236
X_6	$CaCO_3$	-64.18	-32.09	-1.99	0.184
X_7	$MgSO_4{\cdot}7H_2O$	-16.07	-8.04	-0.50	0.667
X_8	KH_2PO_4	-104.39	-52.19	-3.24	0.083
X_9	Sea salt	-61.20	-30.60	-1.90	0.198

R^2 = 96.80%; R^2adj = 95.42%.

Table S8 The dose of important factors in response surface analysis.

Level	Glucose (g/L)	Starch soluble (g/L)	KH_2PO_4 (g/L)
-1	22	5	0.28
0	26	7	0.35
1	30	9	0.42

Table S9 Analysis of variance (ANOVA) for the second-order polynomial model.

Source	DF	SS	*F*-value	*p*-value
Model	9	174647	62.72	0.000
Linear	3	139635	150.43	0.000
Quadratic	3	31432	33.86	0.001
Cross product	3	3581	3.86	0.090
Residual	5	1547		
Lack of Fit	3	1353	4.63	0.183
Pure Error	2	195		
Total	14	176194		

DF: degree of freedom; SS: sum of squares.

Figure S1 Phylogenetic tree of *S. somaliensis* SCSIO ZH66 based on 16S rRNA sequences.

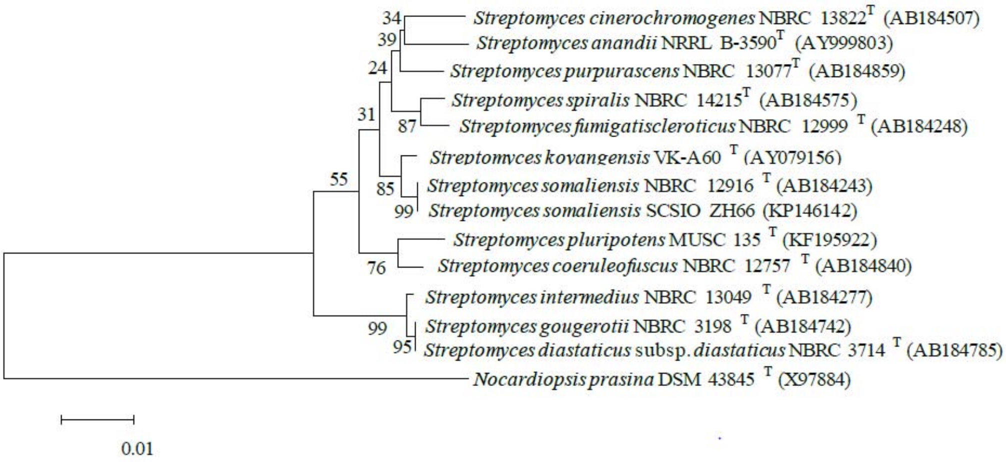

Figure S2 Phenotypes of strain ZH66-WT and strain ZH66-RIF1. (A, B) surface view; (C, D) microscopic analysis. Strains were incubated on Gauze's No. 1 medium for 72 h at 30 ℃. Bar, 5 μm.

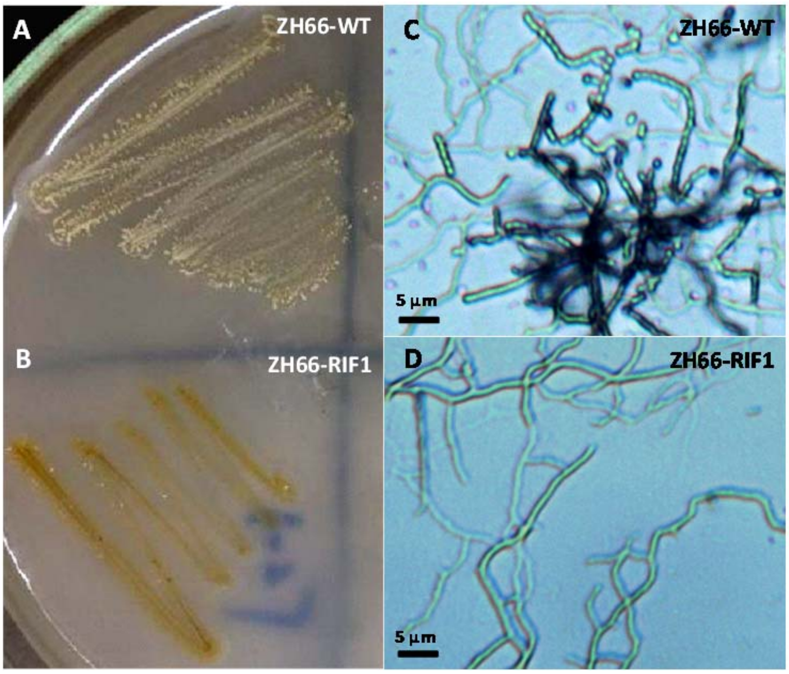

Figure S3 HPLC traces of fermentation products of strain ZH66-RIF1 in different medium.

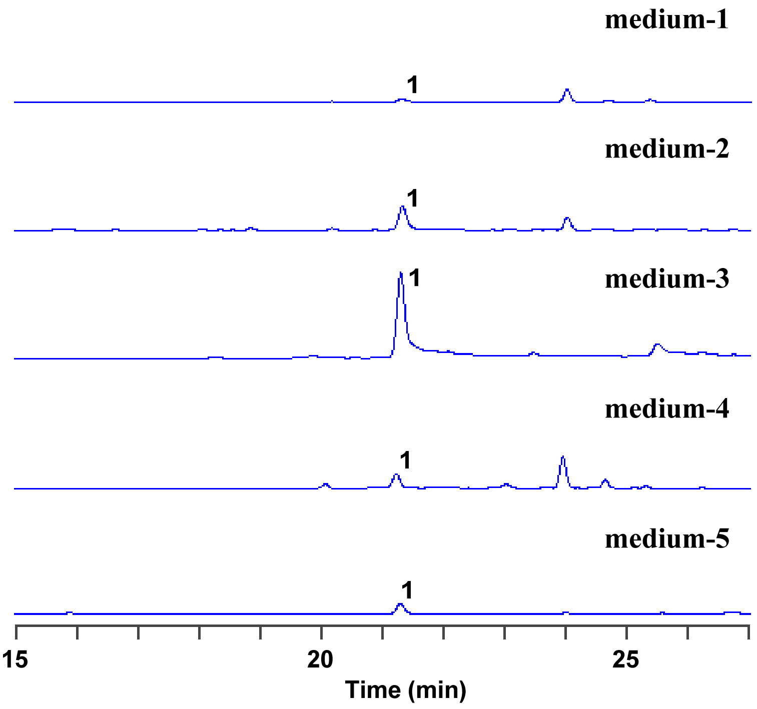

Figure S4 UV spectrum of FDM A.

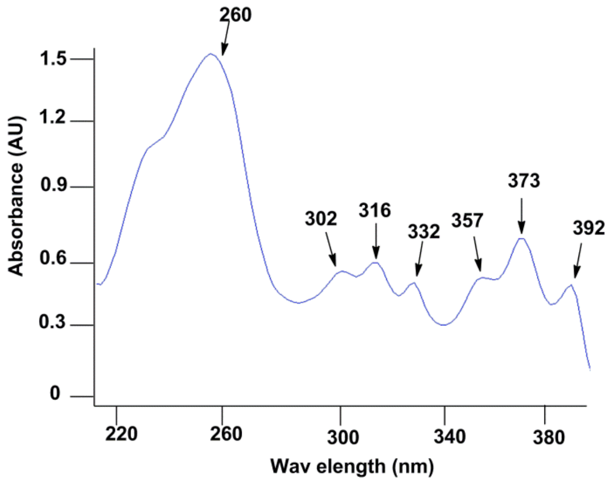

References

1. Wendt-Pienkowski E, Huang Y, Zhang J, Li B, Jiang H, Kwon H, et al. Cloning, sequencing, analysis, and heterologous expression of the fredericamycin biosynthetic gene cluster from *Streptomyces griseus*. J Am Chem Soc. 2005; 127:16442-16452.
2. Hosoya Y, Okamoto S, Muramatsu H, Ochi K. Acquisition of certain streptomycin-resistant (*str*) mutations enhances antibiotic production in bacteria. Antimicrob Agents Chemother. 1998; 42(8):2041-2047.
3. Chen Y, Wendt-Pienkowski E, Shen B. Identification and utility of FdmR1 as a *Streptomyces* antibiotic regulatory protein activator for fredericamycin production in *Streptomyces griseus* ATCC 49344 and heterologous hosts. J Bacteriol. 2008; 190:5587-5596.
